# Assessment of acid-base balance at birth in newborns from diabetic mothers

**Published:** 2014

**Authors:** A Stanescu, SM Stoicescu

**Affiliations:** * ”Dr. I. Cantacuzino” Clinical Hospital, Bucharest, Romania; ** ”Carol Davila” University of Medicine and Pharmacy, Bucharest; Neonatology Department, “Polizu” Clinical Hospital, Bucharest; “Alfred Rusescu” Institute for Mother and Child Care, Bucharest, Romania

**Keywords:** diabetes mellitus, pregnancy, IDM (infant from diabetic mother), cord blood sampling

## Abstract

Newborns from diabetic mothers are more frequently born prematurely, large for the gestational age, with difficult respiratory adaptation and risk of RDS (respiratory distress syndrome) and, subsequently, exposed to a higher risk of perinatal distress, hypoxia, metabolic stress and hematologic alterations. Comparing the status at birth of 120 newborns from mothers with diabetes, with 120 controls from uncomplicated pregnancies, over a period of 4 years, in a specialized tertiary center, no significant differences in the immediate outcome of such newborns and similar incidence of hypoxia at birth were shown, as illustrated by the parameters of the acid-base balance (cord pH, BE and HCO3). However, there are significant differences in the route of delivery, with a predominance of C-section deliveries in the diabetic group (4 out of every 5 cases), which might be an important contribution to the relative good status of these newborns at birth. Although balanced at birth, the newborns from diabetic mothers need intensive monitoring and care in the subsequent hours after birth, for important risks such as hypoglycemic episodes.

## Introduction

Newborns from diabetic mothers (IDM-infant from diabetic mother) are known for the relative high-risk of mortality and morbidity [**1**]. The term “infant from diabetic mother” (IDM) refers to those from pregnancies complicated by diabetes mellitus type 1, type 2, and also gestational diabetes, i.e. any type of glucose intolerance diagnosed for the first time during the current pregnancy [**2**,**3**]. It is estimated that in a population based study, approximately 3-10% of the OGTT (oral glucose tolerance test) assessed pregnancies after 24 weeks of gestation, present abnormal glucose levels. Of these, 80-90% are gestational diabetes, and the minority remaining are chronic diabetes type 1 or 2 incidentally discovered during pregnancy [**2**].

Uncontrolled maternal diabetes, especially during the periconceptional period, is the main etiological factor inducing morbidities in the newborn, from congenital malformations such as cardiac malformations and neural tube defects, to altered fetal growth velocities and modified adaptation mechanisms under the metabolic strain that can induce organ-specific modifications such as the hypertrophic cardiomyopathy of the newborn [**4**]. The onset of the metabolic stress is due to the high glucose levels in the maternal blood, transferring to the fetus and inducing fetal hyperinsulinism [**5**]. The well established high-risk of macrosomia/ large for gestational-age (LGA) fetuses, with estimated fetal growths above the 90th percentile for gestational age-adjusted curves is believed to be insulin-induced, with an incidence of 15-45% in the interval compared to the 5-15% in the general population [**2**,**6**,**7**].

**Fig. 1:**
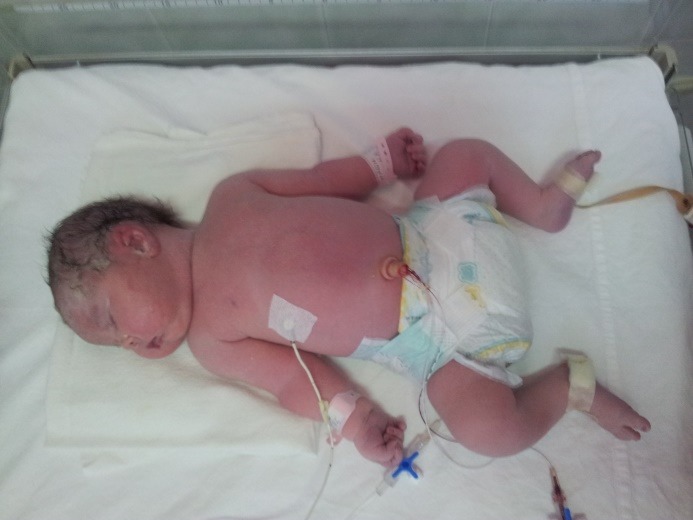
Premature LGA IDM

Newborns from diabetic mothers are more frequently born prematurely, large for the gestational age (**Fig. 1**), but with difficult respiratory adaptation and risk of RDS (respiratory distress syndrome) and, subsequently, exposed to a higher risk of perinatal distress, hypoxia, metabolic stress and hematologic alterations [**1**,**3**]. Most probably, classically described modifications such as myocardial hypertrophy, accelerated growth, polycythemia and modified blood rheology and cerebral perfusion found in the intra-uterine life [**8**] are due to the chronic effects of adaptation mechanisms to the continuous metabolic stress of hyperglycemia [**9**].

Fetal complications add up to the maternal complications triggering a significantly increased risk of premature birth, spontaneous as well as medically planned, in the group of newborns from diabetic mothers compared to the general neonatal population [**10**-**12**].

**Fig. 2:**
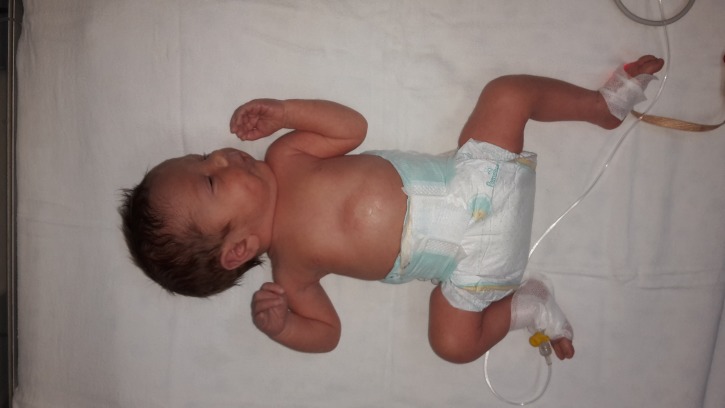
Premature AGA IDM

**The aim of the present study** was to assess the hypoxic perinatal distress of newborns from mothers with diabetes compared to controls from uncomplicated pregnancies, by Apgar score and neonatal metabolic acidosis parameters.

## Material and methods

This was a prospective monocentric study in a tertiary center, the Neonatal Care Unit of “Dr. I. Cantacuzino” Hospital Bucharest, which was in close association with “N. Paulescu” National Institute of Diabetes. The study period was December 2009-December 2013, and included all the neonates from mothers diagnosed with one of the subtypes of diabetes: diabetes type 1, diabetes type 2, and gestational diabetes.

Apart from the epidemiological data, the parameters studied were the following: umbilical blood samples at birth for pH, BE and HCO3, Apgar score. A sample pH below 7,3 and BE below 5mEg/l were considered diagnostic for neonatal hypoxia.

The study group-IDM (infants from diabetes mothers)- with newborns identified from mothers with diabetes was compared to a matched group named non-IDm of controls from age-matched uncomplicated pregnancies. 

Parameters were statistically assessed with a specialized SPSS software vers. 10, and a probability p<0.05 was considered significant. 

## Results

A number of 120 newborns from mothers with diabetes (all types: type 1, type 2 and gestational) have been assessed and compared to 120 age-matched controls born during the same 4-year period. 

Group IDM (n=120): included 30,83% newborns from mothers with diabetes type 1 and 2, from which 31 cases IDD type 1 and 6 cases diabetes type 2. As expected from previous studies, the incidence of gestational diabetes in our group was significantly higher than the pre-existent forms, 69,16% versus 30,83% (p<0,05), ratio 2:1.

**Fig. 3:**
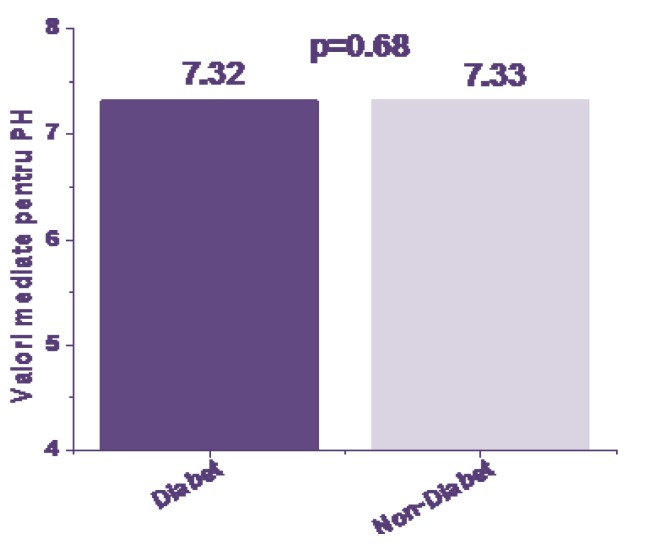
Cord blood pH at birth

**Fig. 4:**
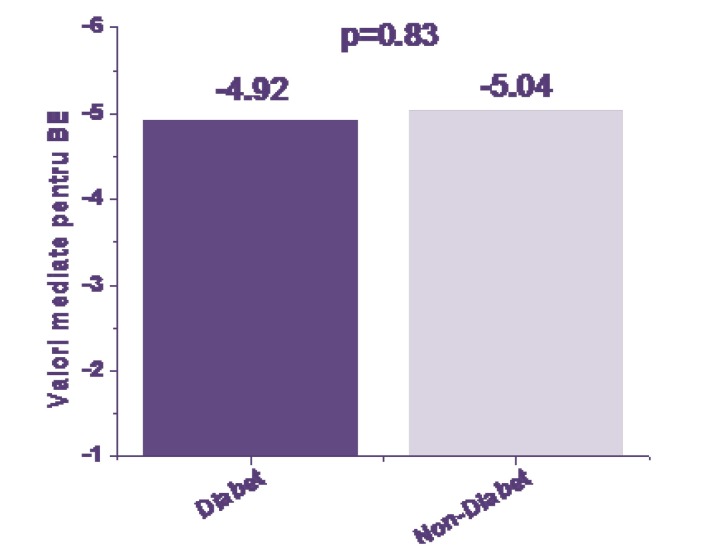
BE at birth

**Fig. 5:**
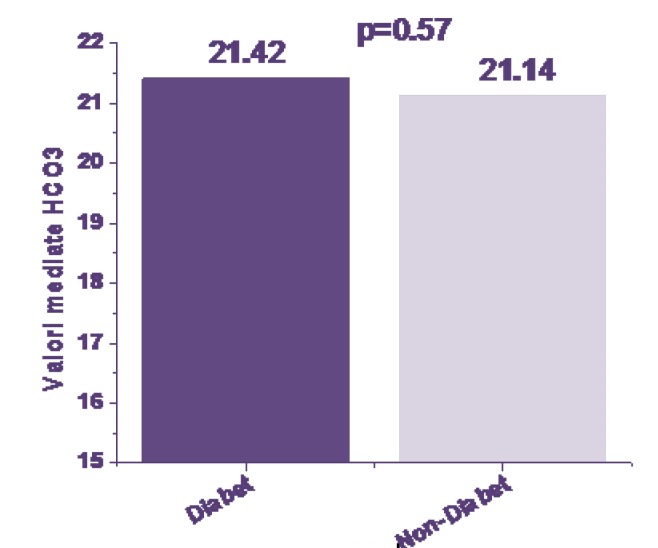
HCO3 at birth

Parameters assessed at birth: average values for umbilical blood pH (**Fig. 3**), BE (**Fig. 4**) and HCO3 (**Fig. 5**) sampled at birth were compared between the groups by using Student t TEst and a probability of <0,05 as threshold for significance. 

From the results, the status at birth from the acid-base balance parameters was not significantly different between the groups. Cord pH values were in average 7,32 for the IDM group versus 7,33 for the non IDM (p=NS). Values of BE were -4,92 for the IDM group versus -5,04 for non-IDM (p=NS). Values HCO3 at birth were 21,42 for the IDM group versus 21,14 for the non IDM (p=NS).

Also, the Apgar score assessment at birth did not show any significant difference between the two groups, with the exception of the subgroup with an Apgar score between 5-7 1 minute after birth: 13 IDM cases versus 8 non-IDM cases, but even in this subgroup the scores became similar at 5 minutes (**Table 1**).

**Table 1:** Apgar score assessment at birth

Apgar score at birth	Apgar 1 min	n	Apgar 5 min	n
IDM	8-10	106	8-10	115
	5-7	13	5-7	5
	4	1	_	_
non IDM	8-9	111	8-10	116
	6-7	8	5-7	4
	4	1	_	_

Type of delivery: Results show a rate of C-sections significantly higher in the IDM group compared to the non-IDM group, respectively 79,15% IDM versus 50% non-IDM (p<0,05). 

**Table 2:** Type of delivery

Group	Type of birth	n
IDM	c- section	95
	spontaneous	25
non IDM	C-section	60
	spontaneous	60

It is important to note that the two groups studied were matched for gestational age at birth, in order to exclude the independent implications of prematurity on the newborn status at birth as a confounding factor. Also, no congenital malformations were identified in the two groups.

## Discussion

This prospective monocentric study demonstrated a prevalence of the incidence of infants from diabetic mothers of 120/ 9057 births over a period of four years, a prevalence of 0,01%, most probably due to the lack of systematic diabetes screening in the general population of our geographical region.

In our experience, the rate of preexistent diabetes cases over the gestational diabetes pregnancies was of 1: 2, in accordance with the previously published data.

When newborns from diabetic mothers were compared at birth with age-matched controls from non-diabetic pregnancies, there were no significant differences of the acid-base balance parameters on cord blood sampling at birth, so no significant incidence of the immediate perinatal hypoxia. What appeared as dramatically different was the C-section rate between the groups, as 4 in 5 children from diabetic mothers, were delivered by C-section, a rate significantly higher than the normal average.

Therefore, a discussion over the optimum route of delivery remains open, as the current data support the choice of the C-section route as a probable way of protection from the adaptive effort during the expulsion of these fragile newborn, especially because the possibilities of the prenatal assessment of the reserves of these children were extremely limited. Classical methods such as cardiotocography before and during labor, or fetal Doppler and biophysical score assessment are unreliable and not predictive for perinatal hypoxia and sudden death in metabolic diseases such as maternal diabetes.

## Conclusion

Comparing the status at birth of newborns from mothers with diabetes with controls from uncomplicated pregnancies showed no significant differences in the incidence of hypoxia at birth, as illustrated by the parameters of the acid-base balance (cord pH, BE and HCO3). However, there are significant differences in the route of delivery, with a predominance of C-section deliveries in the diabetic group that might have an important contribution to the relative good status of these newborns at birth.

The present study brings arguments that birth by C-section reduces the risk of perinatal hypoxia in newborns from diabetic mothers.
